# The role of cluster size and intra-cluster correlations when adjusting for covariates in the analysis of cluster randomised trials

**DOI:** 10.1186/1745-6215-16-S2-P154

**Published:** 2015-11-16

**Authors:** Neil Wright

**Affiliations:** 1 Queen Mary University of London, London, UK

## 

Reports of clinical trials often include adjusted analyses, which incorporate covariate data into the analysis model. Adjusting for covariates can increase the precision of treatment effect estimates and increase the power of statistical tests, without the need to increase sample size. In individually randomised trials, the main reason to adjust for a particular covariate is that it is expected to be strongly associated with the primary outcome. The larger the association between covariate and outcome, the greater the increase in power achieved from an adjusted analysis.

A valid analysis of a cluster randomised trial (CRT) must take into account the clustered structure of the data, for example by using a mixed effects model. The selection of covariates for an adjusted analysis of a CRT is more complicated because covariates exist at both the cluster level and individual level. Further, adjustment for an individual level covariate can affect the residual variance of the outcome at both the cluster and individual levels.

Using results from simulations, and some analytic investigation, I show how the size of clusters and the intra-cluster correlations of the covariate and outcome affect power and precision in adjusted analyses of CRTs using linear mixed effects models. I also consider logistic mixed effects models and show how adjusting for individual level or cluster level covariates affect what treatment effect is being estimated.

